# Supplementary material for: Physics informed neural network for charged particles surrounded by conductive boundaries
Source: Sci Rep. 2023 Aug 28;13:14072. doi: 10.1038/s41598-023-40477-y (PMC10462718; doi:10.1038/s41598-023-40477-y)
Supplement: Supplementary file 1 — Supplementary Information. [file 41598_2023_40477_MOESM1_ESM.pdf]

Supplementary: hyperparameter tuning for PINN and NN

This supplementary document accompanies the main manuscript and contains three tables concerning hyperparameter tuning that are intended to elaborate on how the parameters used in the study were tuned.

In Table 1, the MSE are reported between the predicted potential and the same potential for various hyperparameter values; $\lambda_2 = [0, 0.1, 0.2, 0.3]$, $\lambda_3 = [0, 0.1, 0.2, 0.3, 0.4]$, the number of hidden layers= $[1, 3, 5, 7]$, and the number of neurons per hidden layer= $[10, 30, 50]$. Table 1 indicates that a model with only one hidden layer cannot accurately predict the potential. Similarly, a model with ten neurons per layer would not be effective. Accordingly, Table 2 and Table 3 reported the results for just $[3, 5, 7]$ layers as well as for $[30, 50]$ neurons per layer. To avoid overfitting, we chose $\lambda_4 = 0.0001$.

Unlike Table 1, Table 2 presents not only the MSE but also the $R^2 score$ of the test set. It can be seen in Table 2 that the model with seven layers and 50 neurons per layer performed better when λ_2 and λ_3 are $0.3, 0.3$ and $0.2, 0.4$, respectively.

Also, the best model could estimate both the potential of the train and the test sets as well as the potential of point-charged particles outside of these sets. Consequently, we evaluate the best tuned-PINN model and RF on the extrapolation samples; the results are reported in Table 3.

λ_2	λ_3	MSE_{test} Neurons=10	Neurons=30	Neurons=50	MSE_{test} Neurons=10	Neurons=30	Neurons=50
		**Num of hidden layer:1**			**Num of hidden layer:5**		
	0.0	0.246	0.246	0.246	0.246	0.246	0.246
	0.1	0.162	0.145	0.124	**0.099**	0.145	**0.078**
0.0	0.2	0.191	0.212	0.132	0.136	**0.062**	**0.072**
	0.3	0.175	0.159	0.171	0.14	**0.069**	0.124
	0.4	0.24	0.171	0.18	0.191	**0.064**	**0.076**
	0.0	0.246	0.247	0.247	0.246	0.246	0.246
	0.1	0.222	0.143	0.117	0.107	0.245	0.192
0.1	0.2	0.201	0.165	0.129	0.243	**0.079**	**0.062**
	0.3	0.22	0.159	0.16	0.112	**0.094**	**0.071**
	0.4	0.249	0.177	0.243	0.205	**0.088**	**0.07**
	0.0	0.246	0.246	0.246	0.246	0.246	0.246
	0.1	0.158	0.144	0.154	0.235	0.161	**0.083**
0.2	0.2	0.225	0.174	0.174	0.198	**0.097**	**0.071**
	0.3	0.223	0.143	0.158	0.192	**0.081**	**0.075**
	0.4	0.197	0.185	0.189	0.216	**0.065**	0.118
	0.0	0.246	0.246	0.246	0.246	0.246	0.246
	0.1	0.21	0.132	0.129	0.245	**0.088**	**0.098**
0.3	0.2	0.199	0.139	0.157	0.209	0.117	**0.065**
	0.3	0.216	0.241	0.202	0.182	0.168	**0.08**
	0.4	0.225	0.182	0.196	0.239	**0.083**	**0.097**
		**Num of hidden layer:3**			**Num of hidden layer:7**		
	0.0	0.246	0.246	0.246	0.246	0.246	0.246
	0.1	0.157	0.104	**0.086**	0.244	0.237	0.247
0.0	0.2	0.151	**0.088**	**0.086**	0.238	0.087	0.105
	0.3	0.13	**0.078**	**0.089**	0.244	**0.07**	**0.067**
	0.4	0.202	0.173	0.102	0.188	**0.068**	**0.063**
	0.0	0.246	0.246	0.246	0.246	0.246	0.246
	0.1	**0.099**	**0.089**	0.132	0.244	0.244	0.244
0.1	0.2	0.198	**0.093**	**0.077**	0.23	0.221	**0.069**
	0.3	0.188	**0.088**	**0.083**	0.224	0.223	**0.082**
	0.4	0.262	0.104	**0.079**	0.213	**0.072**	**0.074**
	0.0	0.246	0.246	0.246	0.246	0.246	0.246
	0.1	0.119	0.146	**0.082**	0.244	0.244	0.245
0.2	0.2	0.172	0.09	**0.086**	0.244	0.244	**0.071**
	0.3	0.179	0.084	**0.085**	0.225	0.251	**0.08**
	0.4	0.197	0.185	**0.086**	0.256	0.166	**0.067**
	0.0	0.246	0.246	0.246	0.246	0.246	0.246
	0.1	0.17	0.125	**0.082**	0.244	0.244	0.244
0.3	0.2	0.199	0.117	**0.096**	0.244	**0.083**	**0.08**
	0.3	0.224	0.107	**0.088**	0.202	0.235	**0.069**
	0.4	0.199	0.222	0.152	0.236	**0.097**	**0.075**

Table 1: MSE between the predicted and the exact potential $\phi(x)$ for a different value of λ_2, and λ_3, and the different number of hidden layers, and neurons per hidden layer in PINN for 1000 different sample of the Test set. Here, $\lambda_4 = 0.0001$ is fixed and $\lambda_1 = 1-(\lambda_2+\lambda_3+\lambda_4)$. In this table, the bold number means $MSE < 0.1$.

		Num_{layer}=3		Num_{layer}=5		Num_{layer}=7	
λ_2	λ_3	MSE	R^2	MSE	R^2	MSE	R^2
				Num_{neuron}=30			
0.0	0.0	0.246	0	0.246	0	0.246	0
	0.1	0.104	0.774	0.145	0.365	0.237	-14.7
	0.2	0.088	0.85	0.062	0.889	0.087	0.807
	0.3	0.078	0.826	0.069	0.884	**0.07**	**0.904**
	0.4	0.173	0.551	0.064	0.863	0.068	0.868
0.1	0.0	0.246	0	0.246	0	0.246	0
	0.1	0.089	0.796	0.245	-1478	0.244	0
	0.2	0.093	0.825	0.079	0.852	0.221	-2.40
	0.3	0.088	0.793	0.094	0.714	0.223	0.094
	0.4	0.104	0.719	0.088	0.793	0.072	0.887
0.2	0.0	0.246	0	0.246	0	0.246	0
	0.1	0.146	0.64	0.161	0.566	0.244	0
	0.2	0.09	0.769	0.097	0.75	0.244	-3167
	0.3	0.084	0.819	0.081	0.857	0.251	-2.32
	0.4	0.185	0.521	0.065	0.874	0.166	0.07
0.3	0.0	0.246	0	0.246	0	0.246	0
	0.1	0.125	0.626	0.088	0.747	0.244	0
	0.2	0.117	0.666	0.117	0.689	0.083	0.863
	0.3	0.107	0.67	0.168	0.59	0.235	0
	0.4	0.222	0.25	0.083	0.837	0.097	0.804
				Num_{neuron}=50			
0.0	0.0	0.246	0	0.246	0	0.246	0
	0.1	0.086	0.832	0.078	0.865	0.247	-254
	0.2	0.086	0.838	0.072	0.873	0.105	0.711
	0.3	0.089	0.767	0.124	0.66	0.067	0.875
	0.4	0.102	0.734	0.076	0.846	0.063	0.849
0.1	0.0	0.246	0	0.246	0	0.246	0
	0.1	0.132	0.64	0.192	-0.48	0.244	0
	0.2	0.077	0.85	0.062	0.888	0.069	0.897
	0.3	0.083	0.837	0.071	0.895	0.082	0.774
	0.4	0.079	0.847	0.07	0.887	0.074	0.863
0.2	0.0	0.246	0	0.246	0	0.246	0
	0.1	0.082	0.756	0.083	0.859	0.245	-1774
	0.2	0.086	0.796	0.071	0.88	**0.071**	**0.908**
	0.3	0.085	0.799	0.075	0.888	0.08	0.87
	0.4	0.086	0.8	0.118	0.727	**0.067**	**0.902**
0.3	0.0	0.246	0	0.246	0	0.246	0
	0.1	0.082	0.75	0.098	0.753	0.244	0
	0.2	0.096	0.777	0.065	0.894	0.08	0.798
	0.3	0.088	0.785	0.08	0.834	**0.069**	**0.902**
	0.4	0.152	0.557	0.097	0.818	0.075	0.867

Table 2: MSE, and R^2 score between the predicted and the exact potential $\phi(x)$ for a different value of λ_2, and λ_3, and the different number of hidden layers, and neurons per hidden layer in PINN for 1000 different samples of the Test set. Here, $\lambda_4 = 0.0001$ is fixed and $\lambda_1 = 1 - (\lambda_2 + \lambda_3 + \lambda_4)$. In this table bold numbers show cases with $MSE < 0.1$ and $R^2 score > 0.9$.

λ_2	λ_3	Num_{layer}=3 MSE	Num_{layer}=3 R^2	Num_{layer}=5 MSE	Num_{layer}=5 R^2	Num_{layer}=7 MSE	Num_{layer}=7 R^2
				Num_{neuron}=30			
0.0	0.0	0.281	0	0.281	0	0.281	0
	0.1	0.14	0.71	0.148	0.364	0.275	-15.28
	0.2	0.118	0.764	0.077	0.847	0.126	0.646
	0.3	0.104	0.737	0.09	0.782	0.089	0.835
	0.4	0.208	0.587	0.072	0.842	0.083	0.822
0.1	0.0	0.281	0	0.281	0	0.281	0
	0.1	0.105	0.808	0.28	-1801	0.28	0
	0.2	0.112	0.775	0.09	0.845	0.259	-2.137
	0.3	0.105	0.793	0.123	0.697	0.264	0.07
	0.4	0.116	0.735	0.12	0.684	0.092	0.813
0.2	0.0	0.281	0	0.281	0	0.281	0
	0.1	0.183	0.601	0.201	0.484	0.28	0
	0.2	0.118	0.739	0.114	0.68	0.28	-3620
	0.3	0.095	0.829	0.109	0.799	0.275	-1.82
	0.4	0.209	0.586	0.078	0.851	0.205	0.183
0.3	0.0	0.281	0	0.281	0	0.281	0
	0.1	0.154	0.663	0.105	0.743	0.28	0
	0.2	0.13	0.74	0.139	0.688	0.109	0.725
	0.3	0.143	0.565	0.2	0.604	0.271	0.012
	0.4	0.269	0.356	0.158	0.364	0.118	0.726
				Num_{neuron}=50			
0.0	0.0	0.281	0	0.281	0	0.281	0
	0.1	0.116	0.764	0.112	0.769	0.28	-310
	0.2	0.1	0.837	0.092	0.829	0.135	0.598
	0.3	0.101	0.79	0.145	0.719	0.083	0.843
	0.4	0.117	0.706	0.096	0.801	0.106	0.662
0.1	0.0	0.281	0	0.281	0	0.281	0
	0.1	0.149	0.657	0.227	-0.465	0.28	0
	0.2	0.121	0.658	0.077	0.832	0.09	0.824
	0.3	0.108	0.719	0.096	0.817	0.102	0.734
	0.4	0.117	0.716	0.085	0.843	0.093	0.842
0.2	0.0	0.281	0	0.281	0	0.281	0
	0.1	0.094	0.782	0.112	0.777	0.28	-2164
	0.2	0.114	0.758	0.094	0.785	0.091	0.854
	0.3	0.101	0.762	0.093	0.818	0.104	0.771
	0.4	0.124	0.644	0.143	0.703	0.088	0.849
0.3	0.0	0.281	0	0.281	0	0.281	0
	0.1	0.097	0.772	0.115	0.727	0.28	0
	0.2	0.112	0.768	0.096	0.807	0.167	0.363
	0.3	0.11	0.777	0.113	0.682	**0.089**	**0.851**
	0.4	0.177	0.563	0.114	0.76	0.103	0.741

Table 3: MSE and R^2 score between the predicted and the exact potential $\phi(x)$ for a different value of λ_2, and λ_3, and the different number of hidden layers, and neurons per hidden layer in PINN for 1000 different samples of the Extrapolate set. Here, $\lambda_4 = 0.0001$ is fixed and $\lambda_1 = 1 - (\lambda_2 + \lambda_3 + \lambda_4)$. In this table bold number shows the best hyperparameters for our PINN-based model.
